# Removal of Phosphorus with the Use of Marl and Travertine and Their Thermally Modified Forms—Factors Affecting the Sorption Capacity of Materials and the Kinetics of the Sorption Process

**DOI:** 10.3390/ma16031225

**Published:** 2023-01-31

**Authors:** Sylwia Gubernat, Adam Masłoń, Joanna Czarnota, Piotr Koszelnik, Marcin Chutkowski, Mirosław Tupaj, Justyna Gumieniak, Agnieszka Kramek, Tomasz Galek

**Affiliations:** 1 Doctoral School of Engineering and Technical Sciences at the Rzeszow University of Technology, Powstańców Warszawy 12, 35-959 Rzeszów, Poland; 2 Inżynieria Rzeszów S.A., ul. Podkarpacka 59A, 35-082 Rzeszów, Poland; 3 Department of Environmental and Chemistry Engineering, Rzeszow University of Technology, Powstańców Warszawy 6, 35-959 Rzeszów, Poland; 4 Department of Chemical and Process Engineering, Rzeszow University of Technology, Powstańców Warszawy 6, 35-959 Rzeszów, Poland; 5 Department of Component Manufacturing and Production Organization, Rzeszow University of Technology, ul. Kwiatkowskiego 4, 37-450 Stalowa Wola, Poland; 6 Department of Integrated Design Systems and Tribology, Rzeszow University of Technology, ul. Kwiatkowskiego 4, 37-450 Stalowa Wola, Poland

**Keywords:** wastewater treatment, phosphorus sorption, ecotechnology, reactive materials, adsorption, phosphorus, green chemistry

## Abstract

The paper presents new reactive materials, namely marl and travertine, and their thermal modifications and the Polonite^®^ material, analyzing their phosphorus removal from water and wastewater by sorption. Based on the experimental data, an analysis of the factors influencing the sorption capacity of the materials, such as the material dose, pH of the initial solution, process temperature, surface structure, and morphology, was performed. Adsorption isotherms and maximum sorption capacities were determined with the use of the Langmuir, Freundlich, Langmuir–Freundlich, Tóth, Radke–Praunitz, and Marczewski–Jaroniec models. The kinetics of the phosphorus sorption process of the tested materials were described using reversible and irreversible pseudo-first order, pseudo-second order, and mixed models. The natural materials were the most sensitive to changes in the process conditions, such as temperature and pH. The thermal treatment process stabilizes the marl and travertine towards materials with a more homogeneous surface in terms of energy and structure. The fitted models of the adsorption isotherms and kinetic models allowed for an indication of a possible phosphorus-binding mechanism, as well as the maximum amount of this element that can be retained on the materials’ surface under given conditions—raw marl (43.89 mg P/g), raw travertine (140.48 mg P/g), heated marl (80.44 mg P/g), heated travertine (282.34 mg P/g), and Polonite^®^ (54.33 mg P/g).

## 1. Introduction

Phosphorus is considered a strategic raw material that is necessary to meet the food needs of the world [1,2]. Considering the limited resources of this element, which occur only in the form of minerals such as apatites (igneous rocks) and phosphorites (sedimentary rocks), the direction of the sustainable use of phosphorus in industry and agriculture deserves special attention, which, in a global sense, is part of the policy of effective resource management to guarantee reserves for future generations [3,4,5]. In the case of phosphorus, huge losses occur, starting from the process of its extraction (exploitation of deposits), processing (e.g., in the production of phosphoric acid), and the use of its products in industry and agriculture [6,7]. The constant reduction of phosphorus resources and the lack of control over the circulation of this element leads to unfavorable processes occurring in the environment. One of them is the disturbance of the balance of the production processes and the decomposition of organic substances in water bodies, leading to an increase in the productivity of these waters and their secondary pollution called eutrophication [2,8,9]. One of the main sources of phosphorus is municipal wastewater, providing 50–90% of the phosphorus compounds that are supplied to receivers along with discharged, treated wastewater, which makes it necessary to look for more and more effective technologies for the treatment of wastewater from phosphorus compounds [4,8].

The currently used biological methods of wastewater treatment cause the “eutrophication potential” of the treated wastewater to be high due to the large number of phosphorus forms available to plants in this wastewater, which in turn, will result in further reductions in the permissible concentrations of this element in the treated wastewater [8,10,11]. Additionally, taking into account the enormous importance of phosphorus in the economy and meeting the existential needs of mankind and its limited resources and large losses, apart from phosphorus removal from the treated medium, its recovery and reuse should also be taken into account. Phosphorus can be recovered from wastewater, sewage sludge, and sludge dewatering leachate, as well as from the ashes obtained from the thermal treatment of sewage sludge [4,12]. Although the potential and possibilities of phosphorus recovery from these sources are high, due to the lack of legal regulations regarding the recycling of this element, especially in water and sewage management, the currently used technologies in municipal wastewater treatment plants (chemical precipitation and biological methods) are not adapted for this purpose; the modernization of technological lines in this aspect is economically unjustified, taking into account the prices of agricultural fertilizers [5,13,14]. In addition, the currently used methods of wastewater treatment from phosphorus compounds do not effectively prevent eutrophication. Currently, special attention is focused on technologies that use materials with sorption properties that exhibit a natural ability to bind phosphorus via sorption or precipitation [15]. Due to the specific nature of the adsorption process, i.e., the binding of molecules or ions on the surface or the physical interface, and the reverse process, i.e., desorption, phosphorus sorbents (reactive materials) are a promising and sought-after technology element for the simultaneous treatment of wastewater from phosphorus compounds and its recovery or use as a fertilizer, which fits perfectly with the principles of the circular economy and sustainable development [16,17,18].

The process of adsorption, i.e., the concentration of the substance adsorbed on the surface of the sorbent, is divided into two basic mechanisms: physical adsorption and chemical adsorption. The binding mechanism of molecules or ions depends on the energy of the adsorbent–adsorbate interaction, which is determined by the nature of the adsorbent surface, as well as the structure of the adsorbent molecules [19]. The sorption process is complex and difficult to define unambiguously, therefore adsorption isotherms are determined, i.e., the functional dependence of the degree of adsorbent coverage with adsorbate with equilibrium pressure under isothermal conditions [19,20]. On the basis of the determined and best-fitted isotherms and parameters of the adopted models, it is possible to determine whether the adsorbate bond is physical or chemical, as well as to describe the adsorbent in terms of structural or energy homogeneity [19,20,21]. The empirical equations used to describe the sorption equilibrium in the liquid–solid system are based mainly on the Langmuir isotherm model, which assumes sorption on an energetically homogeneous surface, and the Freundlich model, where the main assumption is to bind the adsorbate on a heterogeneous surface with a different binding energy without achieving the maximum sorption capacity. The use of models combining these two approaches, such as the Langmuir–Freundlich, Tóth, Radke–Praunitz, and Marczewski–Jaroniec models, makes it possible to determine the maximum sorption capacity on a heterogeneous surface [21,22,23].

Due to the indicated adsorption mechanisms, which depend on the physicochemical parameters of the adsorbent, as well as on the surface charge and pore size, individual reactive materials have different equilibrium times. A key factor in assessing the efficiency of an adsorbent material is the adsorption rate, which is determined on the basis of the designated kinetic models describing this sorption process over time. The most commonly used adsorption kinetic models include the pseudo-first-order model proposed by Lagergren [24] and the pseudo-second-order model, determined by Ho [25], while hybrid models combining the advantages of individual models allow for a broader and more accurate analysis of sorption kinetics [26].

So far, well-maintained reactive materials (in terms of phosphorus binding) have been described in detail in a review [17], which presents natural and synthesized materials along with their modifications, waste, and commercialized materials, such as Polonite^®^, which has been thoroughly investigated in this area [27,28,29]. Additionally, the group of phosphorus sorbets includes iron-calcium salt [30], Tunisian activated clays [31], hybrid material of Mg/Al-layered double hydroxide and almond shell biochar [32], biochars [33,34], magnetic La_2_(CO_3_)_3_/CoFe_2_O_4_/biochar [35], and calcium-modified attapulgite [36]. After recognizing their sorption properties in static conditions, the tested reactive materials should be tested in dynamic conditions in order to comprehensively assess the possibility of using them in sewage treatment, the pretreatment of sewage [37], and the treatment of surface water and drains from green roofs or agricultural drains [38]. Nevertheless, in order for the process of sustainable phosphorus management to take place at every stage of its circulation in the environment (sewage, water, and bottom sediments), it is necessary to thoroughly understand the mechanism of phosphorus binding by sorbents. In addition to controlling the eutrophication process, it will be the basis for the synthesis or modification of new reactive materials towards more effective sorbents, for example, by using the synthesis technique and appropriate catalysts [39,40,41]. Due to being limited to basic studies of phosphorus binding in static conditions, the currently available references do not find an answer to the question of how to combine the need to meet the world’s food needs with a reduction in the negative impact of the phosphorus used (to fertilize agricultural fields) on the aquatic environment [2].

Materials included in the area of “green chemistry” are marl and travertine, and their forms are subjected to thermal treatment which has not been tested so far in terms of phosphorus binding [42]. When taking into account the current challenges in the field of ecotechnology, ecotoxicology, and ecohydrology, the paper presents multiaspect studies of selected materials, allowing for a comprehensive analysis and understanding of the phosphorus sorption process through the use of isotherm and kinetic models, which have not been used so far in the aspect of phosphorus binding, which, in combination with other physicochemical studies, materials, and their effectiveness in various process conditions, allow for a credible discussion on the binding mechanism of this element. In addition, the applied approach enables the assessment of a given material in terms of the recovery of adsorbed phosphorus or a form of sorbent with adsorbed phosphorus.

The paper presents research aimed at recognizing the structure of selected reactive materials, i.e., marl and travertine, with their thermal modifications and the Polonite^®^ filter material in the aspect of the phosphorus bonding process on the surface of these materials. The analysis included the determination and fitting of kinetic models and adsorption isotherms, as well as the maximum sorption capacity of the materials and the effects of material dose, the pH of the initial solution, and the process temperature on phosphorus retention capacity.

## 2. Materials and Methods

### 2.1. Reactive Materials

**Figure 1:**
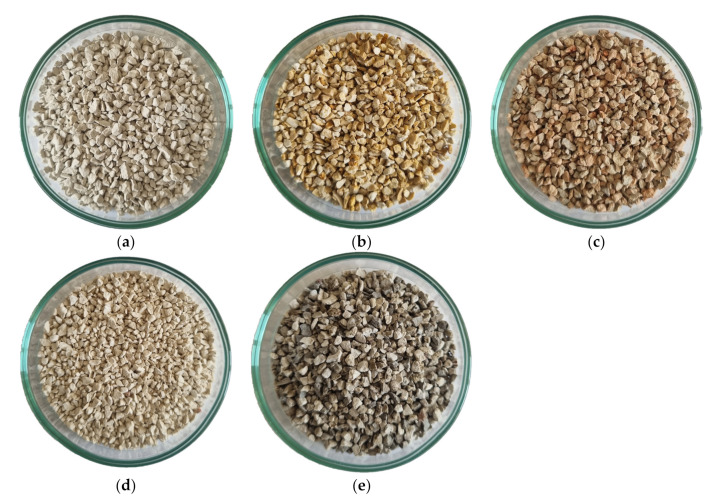
Photographs of tested materials: (**a**) raw marl, (**b**) raw travertine, (**c**) Polonite^®^, (**d**) marl heated at 1000 °C, and (**e**) travertine heated at 700 °C.

The research was carried out using marl, travertine, their thermally modified forms, and Polonite^®^ material (Figure 1). The physicochemical characteristics of the materials are presented in Table 1, while the elemental composition of the sorbents is presented in Table 2.

Marl is a sedimentary, carbonate-clay rock, constituting a transitional rock between clays and limestones. The material for the research was taken from the Lublin Upland in Poland. A fraction of marl with a size of 1–2 mm, a density of 2.76 g/cm^3^, and a specific surface area of 24.41 m^2^/g were used. This material was subjected to heat treatment at 1000 °C in a muffle furnace for 1 h, which resulted in a reduction of its specific surface area while increasing the radius of its pores from 82.81 nm to 93.65 nm.

Travertine is a sedimentary rock that is a concise variety of limestone necrosis, which is formed by precipitation of calcium carbonate from spring waters. The source of travertine (for research) was the mine of this raw material in Raciszyn in Poland, where it takes the form of crystallized rocky limestones with characteristic, irregular cavernousness. A travertine fraction of 1–2 mm with a density of 2.79 g/cm^3^ was used for the study. The material was heat treated at 700 °C in a muffle furnace for 1 h, which increased its specific surface from 0.18 to 0.26 m^2^/g and the radius of its pores from 68.99 to 87.04 nm.

Polonite^®^ is a commercialized filter material used in water and wastewater treatment technology, the main component of which is a heat-treated rock.

### 2.2. Batch Experiments

Studies of the phosphorus adsorption process by selected materials were carried out by a static method, shaking for 24 h and at a rotational speed of 350 rpm for 24 h, a material fraction of about 1–2 mm with 50 mL of KH_2_PO_4_ solution. The tests were carried out at an ambient temperature of 25 °C.

Determination of the effect of the dose of the materials on the phosphorus binding capacity was determined by using the following material doses of 0.5 g, 1 g, 1.5 g, 2 g, and 2.5 g at an initial concentration of the solution of 20.04 mg P/L.

The relationship between the pH of the initial solution (values used 11.76, 3.00) and the effectiveness of phosphorus binding was determined with the use of KH_2_PO_4_ solutions with a concentration of 19.87 mg P/L and 1 g amounts of material fractions of about 1–2 mm.

In addition, the influence of process temperature on phosphorus binding capacity was investigated, and tests were carried out in the above-mentioned range at temperatures of 18 °C and 25 °C.

The determination of phosphorus concentrations was performed by spectrophotometric method using Spectroquant^®^ tests after the prior mineralization of samples. Spectroquant^®^ tests (measuring range: 0.05–5.00 mg P/L; accuracy: ±0.06 mg P/L; confidence: interval P = 95% ± 0.05 mg P/L; coefficient of variation ±0.0093 mg P/L) are based on a reaction in a sulfur solution, where orthophosphate ions react with molybdate ions to form molybdophosphoric acid, which is then reduced with ascorbic acid to phosphomolybdenum blue, which is determined by a spectrophotometer. Three repetitions were performed for each sample. The final result was considered to be the arithmetic mean of those results that did not differ from the value by less than 5%. 

The recognition of the functional groups responsible for the phosphorus adsorption phenomenon present in the materials was carried out using infrared spectroscopy with Fourier transform infrared spectroscopy (FT-IR). The research was carried out using the Nicolet 6700 FT-IR spectrometer (Waltham, MA, USA). An attachment for the transmission method was used. Transmission spectra were recorded in the range of 400–4000 cm^−1^, with a resolution of 4 cm^−1^. Material samples were homogenized in an agate mortar. Using a cylindrical pellet press with a diameter of 13 mm, a pellet was made using potassium bromide KBr as the medium (2 mg sample of material in 300 mg KBr). A pressure of 8 tons was applied. The bands were interpreted on the basis of the available literature data and correlation tables. Multicomponent matching was performed using the OMINIC software (ver. 9.1, Waltham, MA, USA).

Morphological analysis and elemental composition of materials before and after the sorption process were determined by scanning electron microscopy SEM-EDS (scanning electron microscopy—energy dispersive spectroscopy) using the MIRA3 electronic scanning tool by TESCAN (Brno, The Czech Republic), with a Panalytical spectroscopy adapter (EDS) (Malvern, UK). An accelerating voltage of HV = 20 kV and a beam current of 830 µA were used at a working area of FOV = 276.8 µm. Photon energy range on EDS = 10 keV. SEM-EDS is a research method used in material research for the observation, analysis, and characterization of the surface and surface layer of the surface of the tested materials. A specific area of the surface of the test sample is subjected to a scanning, focused, and concentrated electron beam with a specific energy. The primary electron beam penetrates the surface layer of the material and excites it with various signals coming from the tested layer. The excited and analyzed signal of secondary electrons makes it possible to image the observed surface. The induced and analyzed characteristic X-ray radiation allows for determining the elemental composition of the surface layer of the tested object. The depth of signal excitation is related to the depth of penetration of the electron beam into the tested material and depends on the type of material and the beam energy of the primary electron beam.

The specific surface area BET, volume, and radius of the pores were determined by the porosimetric method using the Nova Station A apparatus (Quantachrome Instruments, Boynton Beach, FL, USA). The BET-specific surface area measurement is based on the Brunauer-–Emmett–Teller multilayer adsorption isotherm. The total pore volume and their diameter are determined on the basis of the amount of vapors adsorbed at a relative pressure close to unity, assuming that the pores are filled with liquid adsorbate. The device and the measuring cell were calibrated before starting the assay. Each material sample was degassed under vacuum at 80 °C for 3 h. After cooling down the degassed sample and reweighing, the sample was placed in the measuring port, and the measurement of the amount of adsorbed gas was started, which was the basis for plotting the isotherm and then the capacity of the adsorption monolayer.

The density of materials was determined using the ULTRAPYC 1200e pycnometer (Quantachrome Instruments, Boynton Beach, FL, USA) using the pycnometric method. Immediately prior to the analysis, the samples were conditioned in a vacuum at 80 °C for 3 h. The volume of the test material, placed in the measuring chamber, was determined by compressing ultra-high purity helium at 20 psi, the atoms of which can penetrate into the pores and gaps with a size of up to 0.2 nm, enabling high accuracy of volume measurement.

The sorption capacity of the materials q_t_ (mg P/g) was calculated according to Formula (1):

(1) $$q_{t}=\frac{C_{0}-C_{t}}{M}\cdot V$$

The phosphorus removal efficiency PR (%) was calculated according to Formula (2):

(2) $$\mathrm{PR}\%=\frac{C_{0}-C_{t}}{C_{0}}\cdot 100$$

where C_o_—initial concentration of phosphorus (mg P/L), C_t_—concentration of phosphorus after time t (mg P/L), V—volume of solution (L), and M—mass of material (g).

Adsorption equilibrium isotherms were determined from the shaking results of 50 mL solutions with concentrations of 1.95, 9.54, 20.26, 45.65, 91.31, 188.72, 381.75, 577.20, 865.45, and 1610.00 mg P/L with 1 g of material weight for 24 h at 25 °C. The data were fitted to the Langmuir, Freundlich, Langmuir–Freundlich, Tóth, Radke–Praunitz and Marczewski–Jaroniec models to determine the phosphorus binding mechanism. The equations of the individual models are presented below.

Langmuir sorption isotherm model [23,43]:

(3) $$q_{e,\mathrm{calc}}=\frac{q_{m}{\mathrm{KC}}_{e}}{1+{\mathrm{KC}}_{e}}$$

Langmuir–Freundlich sorption isotherm model [23]:

(4) $$q_{e,\mathrm{calc}}=\frac{q_{m}{\mathrm{KC}}_{e}}{1+{\left({\mathrm{KC}}_{e}\right)}^{n}}$$

Tóth sorption isotherm model [23]:

(5) qe,calc=qmKCe(1+(KCe)n)1n

Freundlich sorption isotherm model [23,44]:

(6) qe,calc= KCe1n

Radke–Praunitz sorption isotherm model [23]:

(7) $$q_{e,\mathrm{calc}}=\frac{q_{m}{\mathrm{KC}}_{e}}{{\left(1+{\mathrm{KC}}_{e}\right)}^{n}}$$

Marczewski–Jaroniec sorption isotherm model [22,23]:

(8) $$q_{e,\mathrm{calc}}=\frac{q_{m}{\left({\mathrm{KC}}_{e}\right)}^{m}}{1+{\left({\mathrm{KC}}_{e}\right)}^{n}}$$

where q_e_—equilibrium concentration of the adsorbed substance, q_m_—maximum sorption capacity, C_e_—equilibrium concentration of the sorbed substance, K—equilibrium constant, n, and m—heterogeneity parameters of the sorbent surface. 

Variable optimization was carried out using the generalized reduced gradient nonlinear solving method, according to the Ladson and Waren algorithm.

In order to evaluate the kinetics of the adsorption process to determine the effect of contact time on adsorption phosphate removal, studies were conducted for a number of time intervals until equilibrium was reached. At 25 °C, 1g weights with 50 mL KH_2_PO_4_ solution with concentrations of 19.46, 19.26, 19.52, 19.26, and 19.79 mg P/L were shaken at 350 rpm for travertine, Polonite^®^, heated marl, marl, and heated travertine, respectively. Samples were taken at intervals of 0.25, 0.5, 1, 2, 3, 5, 8, 9, 10, 11, 12, 13, 14, 16, 18, 20, 21, 22, 23, and 24 h after the start of the reaction. The obtained results were analyzed according to the kinetics equations of pseudo-first-order models (reversible and irreversible), pseudo-second order (reversible and irreversible), mixed reversible 2–1 (second-order sorption process and first-order desorption process), mixed reversible 1–2 (first-order sorption process and second-order desorption process) related to the concentration of the adsorbate in the solid phase. The equations of the individual models are presented below.

Pseudo-first-order reactionary model, irreversible [26]:

(9) $$q_{t}=q_{e}\left(1-e^{-k_{1}t}\right)$$

Pseudo-first-order reaction model, reversible [26]:

(10) $$q_{t}=q_{e}(1-e^{-k_{1}\frac{q_{m}}{q_{t}}t})$$

Pseudo-second-order reactionary model, irreversible [26]:

(11) $$q_{t}=\frac{q_{e}^{2}k_{1}t}{1+k_{1}q_{e}t}$$

Pseudo-second-order reactionary model, reversible [26]:

(12) $$q_{t}=\frac{q_{m}q_{e}\left(e^{{\mathrm{zk}}_{1}t}-1\right)}{q_{m}\left(1+e^{{\mathrm{zk}}_{1}t}\right)-2q_{e}}$$

(13) $$z=\frac{2q_{m}\left(q_{m}-q_{e}\right)}{q_{e}}\;$$

Mixed order reversible model 2–1 (second order sorption process, first order desorption process) [26]:

(14) $$q_{t}=\frac{q_{m}^{2}q_{e}\left(e^{{\mathrm{zk}}_{1}t}-1\right)}{q_{m}^{2}e^{{\mathrm{zk}}_{1}t}-q_{e}^{2}}$$

(15) $$z=\frac{q_{m}^{2}-q_{e}^{2}}{q_{e}}\;$$

Mixed order reversible model 1–2 (first-order sorption process, second-order desorption process) [26]:

(16) $$q_{t}=\frac{q_{m}q_{e}\left(e^{{\mathrm{zk}}_{1}t}-1\right)}{q_{m}\left(e^{{\mathrm{zk}}_{1}t}+1\right)-q_{e}}$$

(17) $$z=\frac{2q_{m}-q_{e}}{q_{e}}\;$$

where q_e_—equilibrium concentration of the adsorbed substance, q_t_—sorption capacity after time t, q_m_—maximum sorption capacity, and k_1_—rate constant.

## 3. Result and Discussion

### 3.1. Factors Determining the Sorption Capacity of the Tested Materials

#### 3.1.1. Doses of the Materials

**Figure 2:**
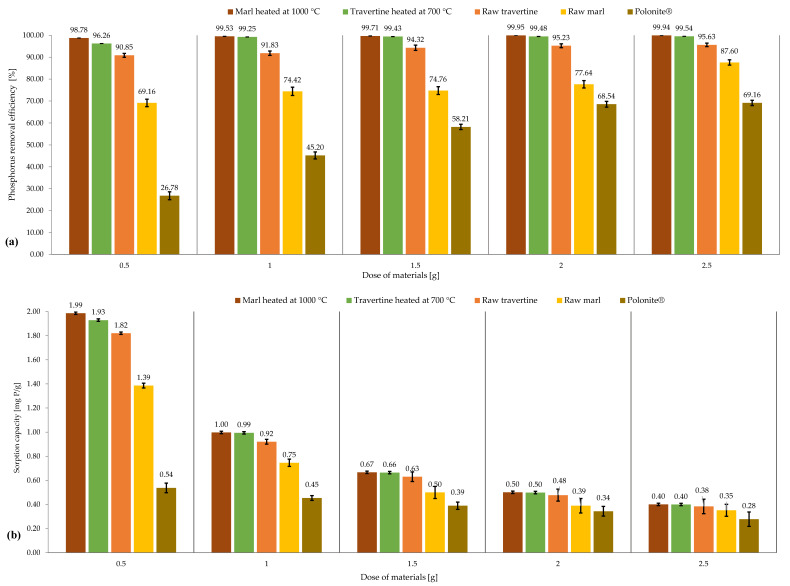
Effect of material dose on (**a**) the efficiency of phosphorus binding and (**b**) sorption capacity.

The results of the studies carried out under different material doses are presented in Figure 2. For all materials, along with an increase in their dose during the process, the efficiency of phosphorus binding increases, which directly results in a decrease in the value of the sorption capacity.

For raw marl, the most pronounced increase in the phosphorus retention efficiency was observed for the dose of 2.5 g, where the efficiency was 87.60%, while for the dose of 2 g, the reduction in phosphorus was 77.64%. For Polonite^®^, the greatest difference was observed for the individual doses. The lowest dose (0.5 g) of this material showed an efficiency of 26.78%, while for the highest dose of 2.5 g, the material achieved a phosphorus reduction of 69.16%. Previous research has shown that for comparable conditions and a dose of 1 g, the efficiency of phosphorus removal for raw marl is 89.98%, and for Polonite^®^, it is 95.09%, which can be interpreted as the instability of the materials due to the heterogeneity of their structure or the chemical compositions of these materials [42].

For the smallest dose, i.e., 0.5 g, raw travertine showed phosphorus retention in the range of 90.85%, while for the largest material input, i.e., 2.5 g, it showed very high efficiency: 95.63%, which is comparable to the effectiveness of travertine heated at the lowest dose.

For heated materials, studies have shown high efficiency in reducing phosphorus (over 99%) in all doses of heated marl, while for heated travertine, this occurs in batch sizes of 1 g, 1.5 g, 2 g, and 2.5 g. The high efficiency of heated marl and travertine, as well as their raw forms, is confirmed by research carried out so far in this aspect [42].

In terms of the sorption capacity of materials, the highest values were achieved for a dose of 0.5 g, respectively, for raw marl: 1.39 mg P/g, raw travertine: 1.93 mg P/g, heated marl: 1.99 mg P/g, heated travertine: 1.93 mg P/g, and Polonite^®^: 0.54 mg P/g.

The improvement of phosphorus binding capacity by increasing the dose of the material results from the larger contact area of the sorbent with the solution, which translates into greater possibilities for sorption processes, such as the adsorption of the adsorbate in pores or through uptake using functional groups. Studies using limestone [45], zeolite [46], akadama clay [47], and dolomite [48] confirm the correlation between the increase in the dose of the material and the increase in the efficiency of phosphorus binding, along with a simultaneous trend of reducing the adsorption capacity per unit mass of the sorbent.

#### 3.1.2. The Initial pH of the Solution

**Figure 3:**
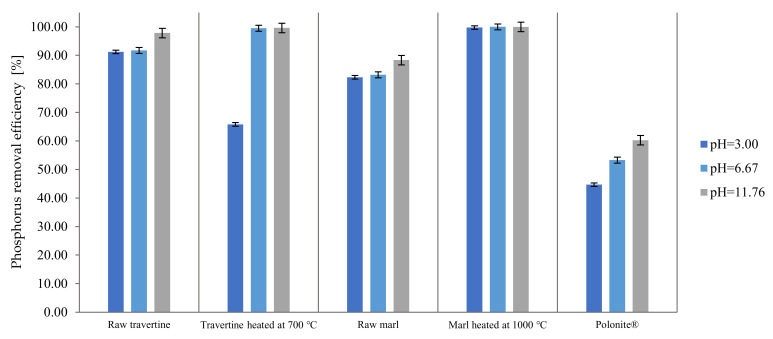
Influence of the pH of the initial solution on the phosphorus retention efficiency of the materials.

The selected materials were also tested at the pH values of the starting solutions (3.00 and 11.76). The results of the research are presented in Figure 3.

Based on the presented results, it can be concluded that the pH of the starting solution has an impact on the efficiency of phosphorus binding by the tested materials. Raw travertine, its heat-treated form, raw marl, and Polonite^®^ at a starting solution pH of 11.76 were more effective than at pH = 3.00. In the case of heat-treated marl, regardless of the pH range of the starting solution, this material showed high efficiency at a level of more than 99%. A clear effect of the low pH of the starting solution on the phosphorus binding process was observed with the use of heated travertine, where a decrease in the phosphorus binding efficiency to 65.82% was noted. The reduction in phosphorus bonding efficiency may result from the instability of travertine that is heat-treated at 700 °C under pH-reduced conditions. The research presented in the paper indicates that this material maintains stability and high efficiency in a pH range above 5.30, while, below this value, there is a decrease in the amount of adsorbed phosphorus. This is confirmed by the literature data, which indicate that materials containing calcium carbonates are more effective in the alkaline pH area, e.g., calcite [49] or marine materials [50,51]. On the other hand, a series of experiments carried out with the participation of various materials showed that as the pH increases, the effectiveness of the process decreases, with examples such as zeolite [46], bauxite [52,53], sepiolite [54], and diatomite [55]. Depending on the content of the individual elements that show a natural ability to bind phosphorus (Fe, Al, Ca, and Mg), the mechanism and effectiveness are also determined by the pH value of the starting solution. For the Al/Fe sorption group, sorption capacity increases in the pH range from neutral to acidic [56], while for the Ca/Mg sorption group, with increasing pH, the efficiency of phosphorus binding increases, which is confirmed by the research presented in this paper.

#### 3.1.3. Process Temperature

**Figure 4:**
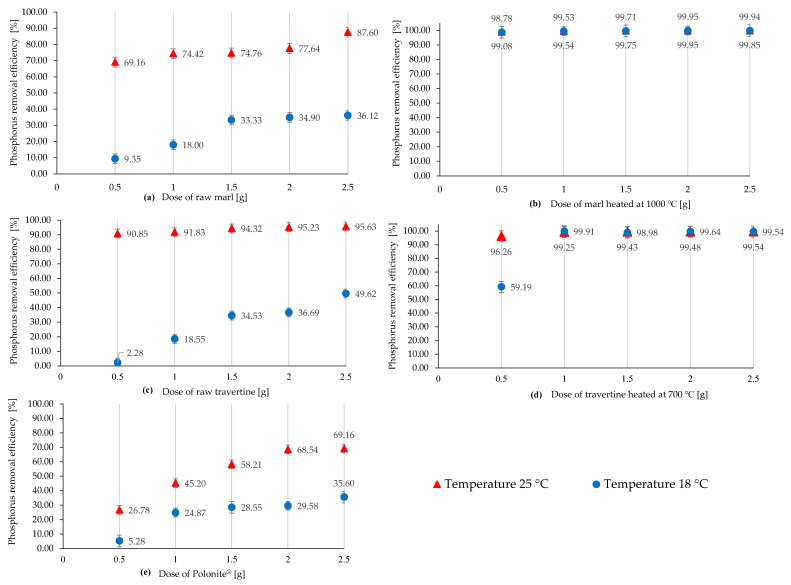
Efficiency of phosphorus removal with different doses at temperatures of 18 °C and 25 °C using (**a**) raw marl, (**b**) heated marl at 1000 °C, (**c**) raw travertine, (**d**) heated travertine at 700 °C, and (**e**) Polonite^®^.

**Figure 5:**
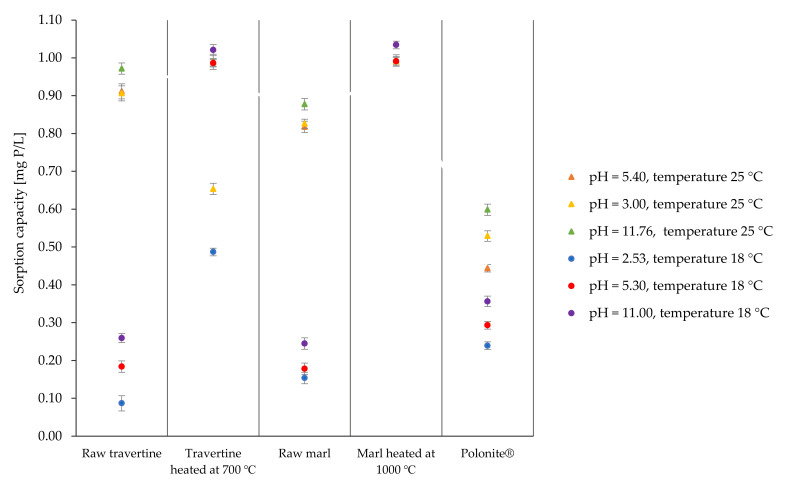
Efficiency of materials at 18 °C and 25 °C for various pH ranges.

When taking into account the occurrence of the variable efficiency of raw materials under similar conditions, all materials were tested in terms of the dose and pH effect on the phosphorus binding process at temperatures of 18 °C and 25 °C; the results are shown in Figure 4 and Figure 5.

For the raw materials and Polonite^®^ (Figure 4a,c,e), it can be observed that at the temperature of 18 °C, the materials show (regardless of the dose) lower phosphorus binding efficiency. Raw travertine (Figure 4c) is sensitive to the process temperature, especially for the lowest doses, where the difference in phosphorus reduction for the 0.5 g dose is 88.57%, and for the 2.5 g dose, the spread of effectiveness is 46.01%. For raw marl, the trend is similar, but the range of differences is clearly smaller; for the indicated doses (0.5 g and 2.5 g), the spread of the phosphorus retention efficiency is 59.81% and 51.48%, respectively. Polonite^®^ is characterized by a lower sensitivity to temperature changes with the use of smaller portions, which increases with increasing dose, according to Figure 4e. Regardless of the temperature, heat-treated marl and travertine (Figure 4b,d) maintain a high phosphorus binding efficiency (over 99%), with the exception of heated travertine (0.5 g), where a decrease in efficiency was noted for the temperature of 18 °C to 59.19%.

Another confirmation of the influence of temperature on the phosphorus sorption process by the reactive materials tested is presented in Figure 5. For the raw materials of marl and travertine, regardless of the pH of the initial solution, the sorption capacity is generally lower at 18 °C than it is at 25 °C. As in the case of the dose, the differences in the sorption capacity of Polonite^®^ for individual temperatures in different pH ranges are not as significant as in the case of marl and raw travertine. Heated marl maintained a high sorption capacity (0.99 mg P/g) regardless of the process conditions. The thermally treated travertine, in the range of the lowest pH values, showed a lower sorption capacity for both temperatures used, but the negative effect on phosphorus retention at lower temperatures was still visible. This was confirmed by the research presented in Figure 5 and the research presented in the previous subsection.

The presented research indicates that the process of phosphorus binding by the tested materials is influenced by temperature. The raw materials and Polonite^®^ especially achieve significantly higher phosphorus binding efficiencies with increasing temperature, which is also confirmed by the literature data for laterite [57], goethite [58], portland cement, fly ash, slag [59], cement [60], and ceramsite [61].

In addition, on the basis of the studies of heat-treated materials, it can be concluded that the heating of materials stabilizes the sorption capacity of the materials and processes occurring during the determination of the adsorbent–adsorbate equilibrium. In the case of marl heated at 1000 °C, it can be considered that the applied roasting temperature of the material is appropriate and has a positive effect on the physicochemical and sorption properties, as manifested by the constant efficiency of this material in the binding of phosphorus in various conditions of the process. In the case of travertine heated at 700° C, it has been shown to have a reduced sorption capacity only in a low pH range, which may indicate that the treatment temperature of 700 °C is not sufficient for the calcination process to proceed in such a way as to allow for the passage of full CaCO_3_ in CaO, which is more reactive to phosphorus [50,62].

In the case of Polonite^®^, according to the research carried out so far, its effectiveness in retaining phosphorus, depending on the process conditions, also varies widely. When considering that it is also a material (opoka) subjected to thermal treatment at 900 °C [63] and that the SiO_2_ content is greater than that of the marl, it may indicate that the thermal treatment of sedimentary rocks (carbonate rocks with clay minerals), together with an increase in the proportion of SiO_2_ and a decrease in the content of calcium, will stabilize the sorption properties less in terms of phosphorus retention. 

#### 3.1.4. Structure and Morphological Analysis of Materials

**Figure 6:**
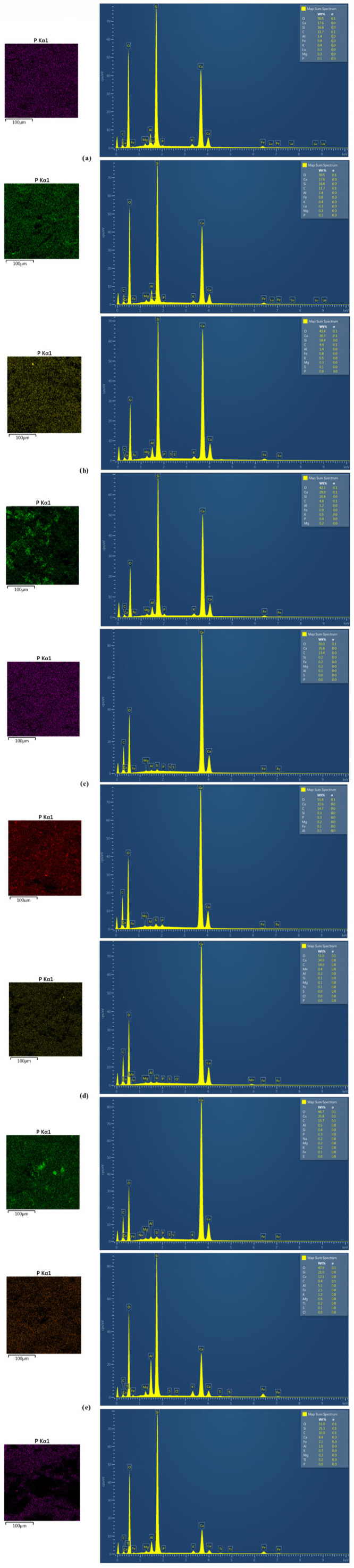
SEM/EDS mapping showing the distribution of phosphorus before and after the sorption process on (**a**) raw marl, (**b**) marl heated at 1000 °C, (**c**) raw travertine, (**d**) travertine heated at 700 °C, and (**e**) Polonite^®^.

SEM-EDS and FT-IR tests were carried out on all materials before and after the sorption process. The results of the research using SEM-EDS are presented in Table 2 and Figure 6. For all materials, the presence of phosphorus after the sorption process was observed, which is revealed both in the elemental composition by increasing its percentage by mass in relation to the raw form and on mapping performed at 1 k magnification. The most noticeable amounts of adsorbed phosphorus are seen in the heat-treated marl (Figure 6b) and travertine (Figure 6d). The largest increase in the amount of phosphorus in the elemental composition of the material was observed in heated marl, which, as a result of the sorption process, increased its content from 0.02% to 0.37%. Bound phosphorus is also visible on natural materials but with a lower density than in the case of thermally modified materials. The Polonite^®^ material was characterized by the weakest increase in the amount of phosphorus on its surface. Thermal modification may improve the sorption properties of the tested materials, which contain all the elements showing the ability to bind phosphorus (Ca, Mg, Al, and Fe) by an increase in the availability of the places where they occur.

**Figure 7:**
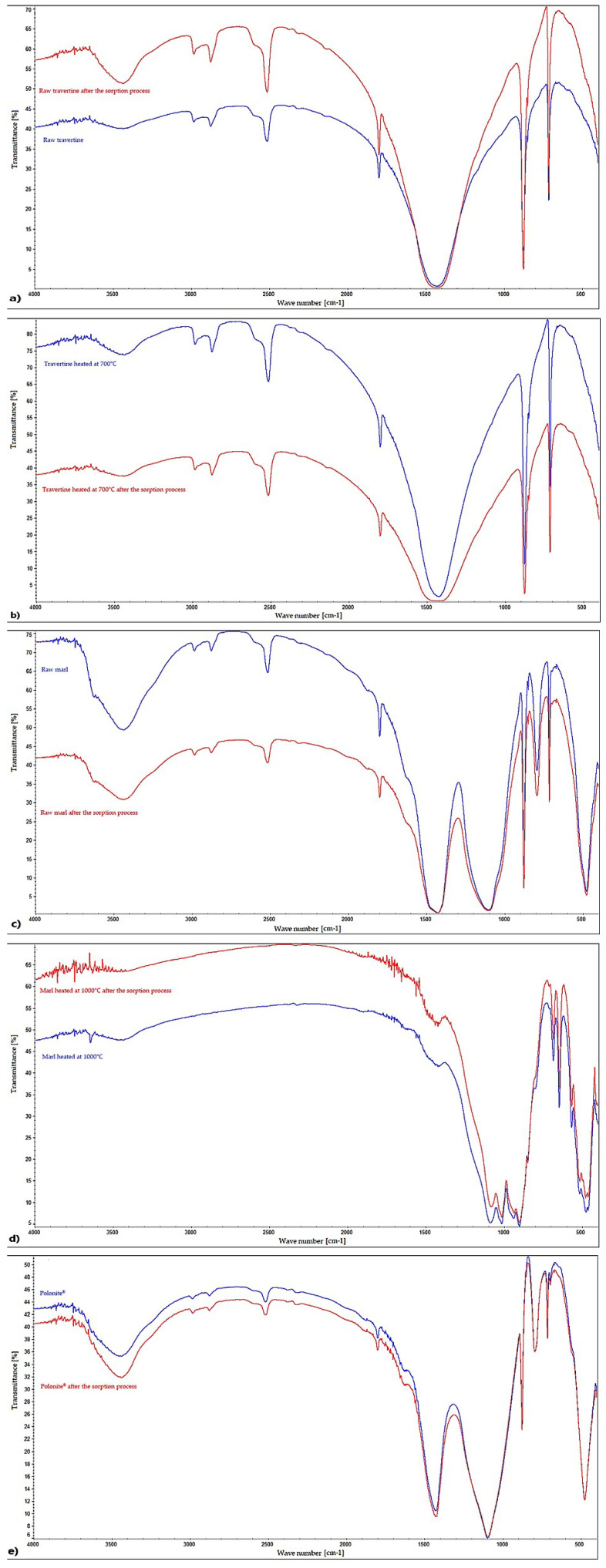
FT-IR spectrum before and after the sorption process of (**a**) raw travertine, (**b**) travertine heated at 700 °C, (**c**) raw marl, (**d**) marl heated at 1000 °C, and (**e**) Polonite^®^.

The materials were subjected to FT-IR tests to identify the functional groups present in the materials and to find the groups responsible for the phosphorus adsorption phenomenon. The results are presented in Figure 7. Raw and heated travertine is characterized by the presence of mainly stretching bonds, C=O, with wave numbers of 2981.34 cm^−1^, 2874.16 cm^−1^, and 1798.09 cm^−1^, describing calcium carbonates, as well as calcite—wave numbers of 1426.80 cm^−1^ and 712.70 cm^−1^ [64,65,66,67,68]. This is confirmed by studies carried out so far on travertine, where it was found that the FT-IR spectrum on this material is a combination of absorption bands occurring for calcite and aragonite [69].

Raw marl (RM), heated marl (HM), and Polonite^®^ (P), in addition to the 1424.74 cm^−1^ (RM), 1420.10 cm^−1^ (HM), and 712.780 cm^−1^ (P) bands corresponding to the tensile bonds characteristic of calcite and carbonates [64,65,66,67,68], also have 875.23 cm^−1^ (RM), 474.85 cm^−1^ (HM), and 474.82 cm^−1^ (P) bands, describing the bonds of Si-O and O-Si-O [70]. The main difference between the raw and heated marl is the disappearance of the hydrogen and carbonate bonds as a result of the heating process (e.g., 2512.94 cm^−1^), which is interpreted as the decomposition of carbonates [71] and the appearance of the 682.49 cm^−1^ band, which is characterized by wollastonite [72]. The indicated types of bindings are confirmed by the literature data from the studies conducted on raw and calcined marl [71,73], as well as on Polonite^®^ material [70,72].

After the sorption process, raw marl, Polonite^®^, raw travertine, and heated travertine did not show the presence of bonds that are characteristic of phosphorus. The reason for this can only be the physical process of phosphorus binding or the overlapping of individual spectra since the most characteristic bands for phosphates appear in the ranges of 460 cm^−1^, 560–600 cm^−1^, 960 cm^−1^, and 1020–1120 cm^−1^ [74], which also occur in the original form of the materials. The only difference is in the magnitude of transmittance intensity. Some authors indicate that a decrease in the intensity of the carbonate anion band may suggest the formation of phosphate complexes, which can be observed in the FT-IR spectra in the case of raw marl, heated travertine, and Polonite^®^ [75].

In the case of heated marl, there are clear differences in the FT-IR spectra before and after the sorption process. There are new bands, such as 1719.37 cm^−1^, characterizing calcium hydrogen phosphate CaHPO_4_, 1644.25 cm^−1^ indicating the presence of hydroxyapatite, and 3589.98 cm^−1^, which may suggest the presence of variscite [76]. Additionally, in addition to the presence of wollastonite, the multicomponent matching indicated the appearance of potassium dihydrogen phosphate on the surface of the thermally modified marl, which allows us to confirm that phosphorus binding on the heated marl surface may take place through a chemical reaction.

### 3.2. Adsorption Isotherms and Determination of the Maximum Sorption Capacity of Materials

**Figure 8:**
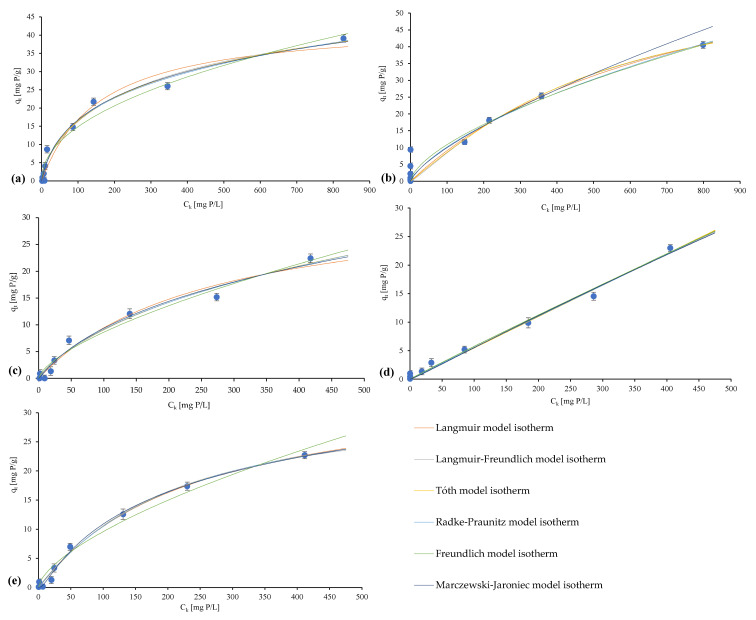
Models of the isotherms for (**a**) raw marl, (**b**) marl heated at 1000 °C, (**c**) raw travertine, (**d**) travertine heated at 700 °C, and (**e**) Polonite^®^.

Adsorption isotherms determined and fitted according to the models of Langmuir, Freundlich, Langmuir–Freundlich, Tóth, Radke–Praunitz, Marczewski–Jaroniec are presented in Figure 8, while the parameters of the individual models are presented in Table 3.

For raw travertine, a coefficient of determination, R^2^, of 0.98 indicates the best fit to the four-parameter Marczewski–Jaroniec model, which characterizes localized physical adsorption, and the values of the heterogeneity parameters n < m indicate the distribution function extended towards low energies. When taking into account a different number of parameters for individual isotherms, the corrected Adj. R^2^ (0.97) shows a best fit to the isotherm from the Freundlich model. The empirical constant n with a value of 1.53 confirms the physical nature of the sorption that takes place favorably, while a 1/n value that is equal to 0.65 informs that the adsorption process takes place on a heterogeneous surface [44,77,78].

The adsorption isotherms determined for heated travertine indicate that the best-fitted model is the Tóth model with a determination coefficient, R^2^*,* of 0.99, while the corrected value of this parameter indicates the Freundlich model. Nevertheless, the fitted models suggest the same nature of the bond—the value of the n parameter in the Freundlich model indicates physical adsorption, and the Tóth model is inherently good at describing this process. In terms of heterogeneity, for the Freundlich model, the value of the heterogeneity parameter 1/n still remains below 1, but a value of 0.96 indicates an increase in this parameter compared to raw travertine, which confirms that the heating process contributes to their stabilization towards an energy that is homogeneous or more homogeneous structurally to a sorbent [44,78,79].

The results for raw marl allowed us to determine the coefficient of determination, R^2^, and its corrected value (0.97 and 0.96, respectively), which indicate the best fit to the Langmuir model, which assumes that the adsorbed molecules form a monomolecular layer, with no interactions between them. The remaining models do not confirm the homogeneity of the adsorbent; for the Tóth and Langmuir–Freundlich models, the heterogeneity parameter n is below 1, which proves the heterogeneity of the sorbent and possible heterogeneity of the active sites [43,79,80].

The process of phosphorus binding by heated marl, similar to its raw form, shows the closest fit to the isotherm determined according to the Langmuir model. In terms of heterogeneity, for the heat-treated form, the n parameters for the Tóth and Langmuir–Freundlich models indicate greater homogeneity of the adsorbent compared to the raw form, as is the 1/n heterogeneity parameter of the Freundlich model, for which an increase to 0.63 was observed, which also confirms the stabilization of the material by thermal treatment, directly reflected in the increased efficiency of phosphorus binding by the heated forms. Additionally, the Marczewski–Jaroniec model, along with the heterogeneity parameters with n > m values, indicate that the distribution function extended towards high energies, whereas, for the raw form of marl, it was found to stretch towards low energies. The nature of the bond in the case of heated marl may have a chemical form because the Langmuir model describes well the monomolecular chemical adsorption, which is confirmed by the presented parameters of individual models [44,80,81].

On the basis of the determined isotherms, the commercialized Polonite^®^ material shows the best fit to the Marczewski–Jaroniec model, while the remaining models and their heterogeneity parameters (due to the values of approximately 1 (Tóth and Langmuir-Freundlich models)) may indicate the structural or energetic homogeneity of this sorbent [43,79].

On the basis of the best-matched isotherms, the values of the maximum sorption capacity of the materials were determined, which are presented in Table 4 in combination with the other materials studied so far in the aspect of phosphorus binding. For raw travertine, q_m_ was achieved at the level of 140.48 mg P/g, while its thermally treated form has a maximum sorption capacity of 282.34 mg P/g. Raw marl has a maximum sorption capacity of 43.89 mg P/g, and its thermally modified form has a maximum sorption capacity of 80.44 mg P/g. A clear increase in the maximum sorption capacity of the materials subjected to thermal treatment confirms the positive effect of the thermal modification of the tested materials on the phosphorus binding process and the unequivocal increase in the actual efficiency of phosphorus reduction (up to 99% efficiency) confirms the necessity to carry it out.

Commercial Polonite^®^ is characterized by a maximum sorption capacity at the level of 54.33 mg P/g (Marczewski–Jaroniec model), which can be confirmed by the literature data, indicating the best fit for the Langmuir model and a sorption capacity at the level of 40.90 mg P/g [82], whereas, for the results for this model presented in the paper (R^2^ = 0.97), the value of the maximum sorption capacity was 36.26 mg P/g.

In comparison with other materials (Table 4), the materials presented in the work, both natural and modified, show a very high phosphorus sorption capacity. In the field of natural materials, similarly high values for raw travertine (140.48 mg P/g) are rarely achieved: an example is Jebel Haidoudi clay (133.88 mg P/g) or Douiret clay (129.30 mg P/g) [31]. In turn, when comparing the values of the maximum sorption capacities of carbonate materials, such as calcite (40.65 mg P/g) [83] and limestone (1.09 mg P/g) [84] with raw travertine, it can be concluded that this material is distinguished by a high capacity, which was determined for the isotherm model according to Marczewski–Jaroniec, which also confirms the need to extend the scope of the modeling of the sorption process for potential phosphorus sorbents with further models (e.g., such as presented in this paper); this fit, apart from determining this parameter, also allowed us to define the sorption mechanism in more detail. A significant increase in the maximum sorption capacity as a result of thermal treatment (of both marl and travertine compared to diatomite (10.2 mg P/g) and its modification (37.3 mg P/g) [55]) also confirms the validity of the implementation of subsequent models for the analyses, especially considering the possible change in the nature of the bond resulting from the modification, as presented in the case of marl and travertine and their thermally treated forms.

### 3.3. Adsorption kinetics

**Figure 9:**
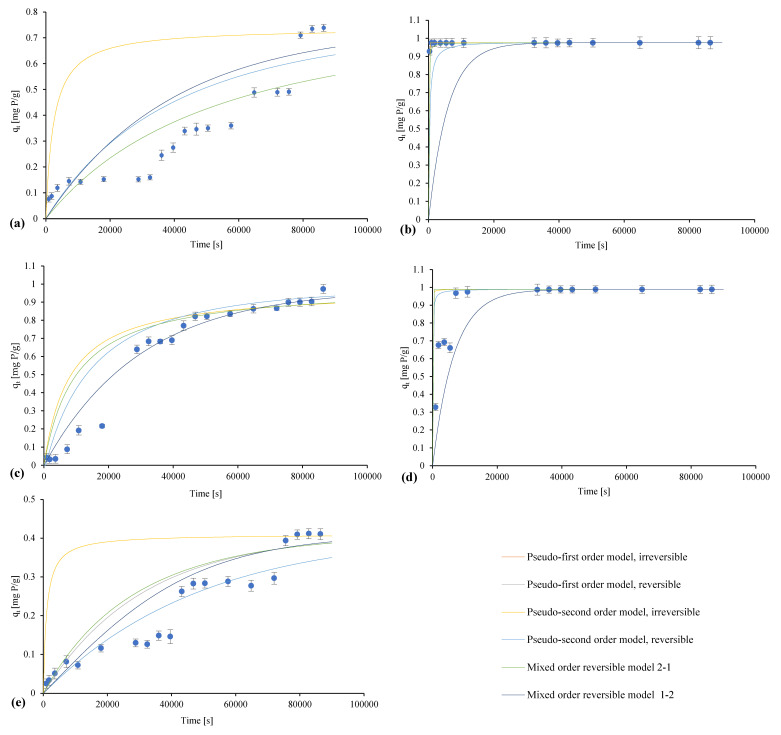
Kinetic models for (**a**) raw marl, (**b**) marl heated at 1000 °C, (**c**) raw travertine, (**d**) travertine heated at 700 °C, and (**e**) Polonite^®^.

The performed kinetic studies aimed to assess the speed and nature of the phosphorus adsorption process in the tested materials, and this information is presented in Table 5 and Figure 9.

Natural marl and travertine, as well as Polonite^®^, are characterized by the slow immobilization of phosphorus on their surface. The phosphorus binding process of these materials can be divided into three stages, the first of which is slow adsorption, which appears as a slow saturation of the pore/functional groups surfaces with phosphorus. The sorbent gradually reaches saturation, and then, after 8 h (travertine), 16 h (marl), 20 h (Polonite^®^), the adsorption process accelerates, which is revealed by a greater drop in phosphorus concentration compared to the first stage. The final phase is the establishment of an equilibrium. The unusual nature of the binding process may be caused by limiting factors, such as the specific structure of the adsorbent, the solution, and its ions [97].

Among the determined kinetic models for raw travertine, the mixed reversible model 1–2 (first-order sorption process, second-order desorption process) with a determination coefficient, R^2^, of 0.97, which shows that the process may be physical, with sorption and desorption occurring, turned out to be the best fit. In kinetic studies, natural marl showed the best fit for the mixed 2–1 reversible model (second-order sorption process, first-order desorption process), which also means that the phosphorus retention process takes place through physical bonding, similar to Polonite^®^, for which (on the basis of the tests carried out under kinetic conditions) it was shown that it is characterized by a best fit to the pseudo-second-order reversible reaction model.

In the case of the heated materials—marl and travertine—the phosphorus binding process over time is characterized by two stages. The first is the very rapid reduction in phosphorus concentration in the solution by saturating the surface with its particles. The second is the saturation of the remaining places until equilibrium is achieved. For the determined kinetic models for heated marl, the pseudo-second-order irreversible model with a coefficient of determination (R^2^) of 0.89 turned out to be the best fit. In contrast, for the heat-treated travertine, the most fitting model was the pseudo-second-order reversible model, with a determination coefficient (R^2^) of 0.87. For heated marl, the reaction rate is higher because the equilibrium is established after 0.25 h, while for heated travertine, it takes place after only 2 h, which is confirmed by the highest value of the k_1_ parameter: equal to 0.96 g/(mg P·min). For the heated travertine, this parameter was 0.02 g/(mg P·min), which may prove the occurrence of a chemical reaction in the case of heated marl.

The characteristics of the individual models correlate with the features of the process established on the basis of the determined isotherms. For heated marl, the irreversible process indicates chemical adsorption, as does the chemical bond of phosphorus on a homogeneous surface. In the case of raw and thermally modified travertine, as well as Polonite^®^, the kinetic tests confirmed the physical nature of the process. Based on the determined isotherms, raw marl showed the best fit to the model describing the chemical process, but at the same time, this indicated the heterogeneity of the surface, which, in combination with the best-suited mixed model characterizing the sorption and desorption processes taking place, may indicate that both physical and chemical processes occur, meaning they can interact directly and limit themselves depending on the conditions, which may explain the unconventional phosphorus binding process over time. In the case of raw travertine, the process of thermal modification probably unblocked the porous structures, and thus, deeper capillaries, which, due to the thermal decomposition of organic substances, became more easily accessible, and transport to the active sites were significantly simplified. Both in the case of marl and travertine, the observation of the differences in the binding of heated and raw forms clearly indicates that in terms of phosphorus binding, thermal treatment improves the sorption properties.

In the studies conducted so far on the kinetics of reactive materials, the most frequently fitted kinetic model was the pseudo-second-order model proposed by Ho, which assumes that the rate-limiting step of the process most likely involves chemical interactions leading to the binding of metal ions or organic compounds on the adsorbent surface via the mechanism (among others) of ion exchange or complexation [25]. The tested sorbents showed the best fit in terms of phosphorus binding to this model via the value of the determination coefficient (R^2^): diatomite (0.99), zeolite (0.97), hydrotalcite (0.98), activated alumina (0.99) [78], hydroxy-aluminum pillared bentonite (1.00), hydroxy-iron pillared bentonite (0.99), mixed hydroxy-iron–aluminum pillared bentonite (1.00) [98], akadama clay (0.99), acid-activated akadama clay (0.99) [47], thermally modified zeolite (0.99) [99], Absodan Plus^®^ (0.99) [97], and Tunisian activated clays (0.99) [31]. A special case was calcium-rich sepiolite, which was characterized by fitting both to the pseudo-first and pseudo-second order kinetics models, which may indicate chemical sorption with valence forces via sharing, i.e., electron exchange between the adsorbent and the adsorbate [54]. Modeling the kinetics of the sorption process using the models presented in the paper (including reversible, irreversible, and mixed models) allows for a broad analysis of phosphorus binding over time and can also confirm the conclusions drawn from the best-fit isotherm models. In addition, the recognition of the nature of the desorption process allowed us (in the subsequent stages) to assess the release of phosphorus from the surface of these materials, which is of decisive importance in the recovery of this element and its reuse.

## 4. Conclusions

The reactive materials presented and used in this work have the ability to retain phosphorus on their surface. All materials increased their phosphorus retention efficiency with increasing doses. Raw marl and travertine turned out to be sensitive to changes in the initial solution pH and the temperature of the process. The thermal treatment of marl and travertine stabilizes them in terms of their sensitivity to changing conditions, such as temperature and pH, and increases the efficiency of phosphorus removal. Each material has a different surface in terms of structure and energy, as confirmed by the tailored adsorption isothermal models and kinetic models. Raw marl, raw travertine, and Polonite^®^ probably retain phosphorus by physical bonding, with the indication in the case of raw marl being the simultaneous physical and chemical processes. The binding of phosphorus by materials subjected to heat treatment on the basis of the fitted models and FT-IR test spectra indicate chemical binding in the case of heated marl and physical binding in heated travertine.

## Figures and Tables

**Table 1:** Physicochemical properties of materials.

Material	Fraction [mm]	Density [g/cm^3^]	Specific Surface Area BET [m^2^/g]	Total Pore Volume [cm^3^/g]	Average Pore Radius [Å]
**Raw travertine**	1–2	2.79	0.18	0.0013	1380
**Raw marl**	1–2	2.76	24.41	0.2021	1660
**Travertine heated at 700 °C**	1–2	2.72	0.26	0.0022	1740
**Marl heated at 1000 °C**	1–2	2.89	0.91	0.0085	1870
**Polonite^®^**	1–2	2.53	10.62	0.0626	1180

**Table 2:** Elemental composition of materials.

Material	Elemental Composition [%]												
	C	O	Na	Mg	Al	Si	P	S	Cl	K	Ca	Ti	Fe
**Raw travertine**	13.36	50.01	-	0.18	0.14	0.22	0.01	0.03	-	-	35.84	-	0.21
**Travertine heated at 700 °C**	14.05	50.99	-	0.12	0.18	0.12	0.01	0.02	0.02	-	33.98	-	0.09
**Raw marl**	14.59	53.73	0.05	0.24	1.15	14.88	-	-	-	0.28	14.62	-	0.47
**Marl heated at 1000 °C**	4.4	43.42	-	0.33	1.42	18.44	0.02	0.06		0.49	30.66	-	0.76
**Polonite^®^**	9.36	47.88	-	0.62	5.13	20.97	-	0.06	0.04	1.16	12.07	0.23	2.47

**Table 3:** Values of parameters of the isotherm models for the materials.

Isotherm Model	Model Parameters		Raw Travertine	Travertine Heated at 700 °C	Raw Marl	Marl Heated at 1000 °C	Polonite^®^
Langmuir	q_m_	[mg P/g]	34.13	4314.61	43.89	80.44	36.26
	K	[L/mg]	3.83 × 10^−3^	1.28 × 10^−5^	6.23 × 10^−3^	1.28 × 10^−3^	4.06 × 10^−3^
	R^2^	[-]	0.973	0.993	0.968	0.955	0.992
	Adj R^2^	[-]	0.969	0.992	0.964	0.949	0.991
Freundlich	K	[mg P/g]	0.42	0.07	1.71	0.59	0.50
	n	[-]	1.53	1.04	2.13	1.58	1.56
	R^2^	[-]	0.975	0.992	0.944	0.885	0.980
	Adj R^2^	[-]	0.971	0.991	0.936	0.869	0.977
Langmuir–Freundlich	q_m_	[mg P/g]	59.32	536.03	61.34	65.09	32.52
	K	[-]	1.19 × 10^−3^	1.11 × 10^−4^	2.42 × 10^−3^	1.93 × 10^−3^	5.11 × 10^−3^
	n	[-]	0.80	1.01	0.71	1.16	1.09
	R^2^	[-]	0.976	0.992	0.953	0.895	0.993
	Adj R^2^	[-]	0.968	0.990	0.938	0.860	0.990
Tóth	q_m_	[mg P/g]	125.35	282.34	92.25	57.03	33.28
	K	[-]	1.90 × 10^−3^	1.95 × 10^−4^	9.89 × 10^−3^	1.57 × 10^−3^	4.20 × 10^−3^
	n	[-]	0.42	3.88	0.40	1.56	1.12
	R^2^	[-]	0.977	0.993	0.957	0.895	0.992
	Adj R^2^	[-]	0.969	0.991	0.943	0.860	0.990
Radke–Praunitz	q_m_	[mg P/g]	17.12	15.65	15.82	9.31	37.69
	K	[-]	1.11 × 10^−2^	7.11 × 10^−3^	3.85 × 10^−2^	2.89 × 10^−2^	3.78 × 10^−3^
	n	[-]	0.65	0.03	0.72	0.46	1.05
	R^2^	[-]	0.977	0.993	0.960	0.886	0.992
	Adj R^2^	[-]	0.970	0.991	0.946	0.848	0.990
Marczewski–Jaroniec	q_m_	[mg P/g]	140.48	12309.86	123.92	121.63	54.33
	K	[-]	1.91 × 10^−1^	4.17 × 10^−5^	7.94	3.17 × 10^−4^	2.71 × 10
	n	[-]	0.29	0.28	0.26	4.89	0.43
	m	[-]	2.25	1.25	3.36	0.72	21.23
	R^2^	[-]	0.978	0.992	0.960	0.891	0.995
	Adj R^2^	[-]	0.964	0.987	0.936	0.826	0.992

**Table 4:** Comparison of different materials in terms of phosphorus adsorption capacity.

Material	Adsorption Isotherm Parameters According to Langmuir Model * Marczewski–Jaroniec Model ** Tóth Model		Literature
	q_m_ [mg P/g]	R^2^ [-]	
limestone	1.09	-	[84]
calcite	40.65	0.99	[83]
goethite	27.00	0.99	[58]
apatite	4.76	0.97	[85]
opoka heated at 900 °C	181.82	0.99	[86]
travertine	140.48 *	0.98	own research
travertine heated at 700 °C	282.34 **	0.99	own research
marl	43.89	0.97	own research
marl heated at 1000 °C	80.44	0.95	own research
shale	0.65	-	[87]
laterite	1.14	0.99	[57]
acadama clay	6.09	0.96	[47]
diatomite	10.2	0.99	[55]
diatomite modified with ferrihydrite	37.3	0.99	[55]
powdered ceramsite	0.59	0.99	[88]
brick dust	0.46	0.99	[88]
lanthanum III modified bentonite	14.00	0.99	[89]
biochar	2.39	0.96	[90]
Jebel Haidoudi clay	133.88	0.99	[31]
Douiret clay	129.30	0.96	[31]
biochar with dolomite	29.18	0.98	[91]
coal slag	21.63	0.51	[92]
autoclaved concrete	70.90	0.94	[93]
red mud	113.87	0.96	[94]
fly ash	63.22	0.99	[94]
Pollytag^®^	32.24	0.93	[95]
Polonite^®^	40.90	-	[82]
Polonite^®^	36.26	0.97	own research
	54.33 *	0.99	
Leca^®^	5.10	-	[82]
Rockfos^®^	256.4	0.99	[96]

**Table 5:** Values of parameters of isotherm models for materials.

Kinetic Model	Model Parameters		Raw Travertine	Travertine Heated at 700 ℃	Raw Marl	Marl Heated at 1000 ℃	Polonite^®^
Pseudo-first-order model, irreversible	R^2^	[-]	0.9703	0.8436	0.7240	0.3092	0.8079
	k_1_	[1/min]	3.35 × 10^−5^	1.51 × 10^−4^	2.55 × 10^−5^	1.70 × 10^−4^	3.23 × 10^−5^
Pseudo-first-order model, reversible	R^2^	[-]	0.9703	0.8436	0.7240	0.3092	0.8079
	k_1_	[1/min]	3.30 × 10^−5^	1.50 × 10^−4^	2.20 × 10^−5^	1.69 × 10^−4^	2.92 × 10^−5^
Pseudo-second-order model, irreversible	R^2^	[-]	0.8589	0.8710	0.3643	0.8862	0.4144
	k_1_	[g/(mg P·min)]	1.29 × 10^−4^	1.17 × 10^−1^	5.59 × 10^−4^	9.57 × 10^−1^	2.39 × 10^−3^
Pseudo-second-order model, reversible	R^2^	[-]	0.9242	0.8724	0.7189	0.7732	0.8748
	k_1_	[g/(mg P·min)]	5.00 × 10^−5^	1.78 × 10^−2^	1.66 × 10^−5^	3.63 × 10^−3^	1.00 × 10^−5^
Mixed order reversible model 2–1	R^2^	[-]	0.8855	0.8722	0.7522	0.8715	0.7923
	k_1_	[g/(mg P·min)]	9.96 × 10^−5^	1.90 × 10^−2^	1.36 × 10^−5^	2.89 × 10^−2^	1.34 × 10^−5^
Mixed order reversible model 1–2	R^2^	[-]	0.97034	0.84366	0.72401	0.30914	0.84910
	k_1_	[g/(mg P·min)]	3.35 × 10^−5^	1.51 × 10^−4^	2.55 × 10^−5^	1.70 × 10^−4^	1.00 × 10^−6^

## Data Availability

Not applicable.

## Nomenclature

**Parameters**	**Description**	**Unit**
**PR (%)**	phosphorus removal efficiency	[%]
**q_e,calc_**	equilibrium concentration of the adsorbed substance predicted by the isotherm model	[mg P/g]
**q_e_**	equilibrium concentration of the adsorbed substance	[mg P/g]
**q_m_**	maximum sorption capacity	[mg P/g]
**q_t_**	sorption capacity after time t	[mg P/g]
**C_e_**	equilibrium concentration of the sorbed substance	[mg P/L]
**K**	equilibrium constant	[-]
**n, m**	heterogeneity parameters of the sorbent surface	[-]
**t**	time	[s]
**C_o_**	initial concentration of phosphorus	[mg P/L]
**C_t_**	concentration of phosphorus after time t	[mg P/L]
**V**	volume of solution	[L]
**M**	mass of material	[g]
**k_1_**	rate constant	[1/min], [g/(mg P·min)]
**R^2^**	coefficient of determination	[-]
**Adj R^2^**	adjusted coefficient of determination	[-]
**z**	corresponding variable	[-]
**Abbreviation**	**Description**	
SEM	scanning electron microscopy	
SEM-EDS	scanning electron microscopy—energy dispersive spectroscopy	
BET	Brunauer, Emmett, and Teller (the method of determination of the specific surface area)	
FT-IR	Fourier transform infrared spectroscopy	
RM	raw marl	
HM	heated marl at 1000℃	
P	Polonite^®^	
